# Protocol for a Randomised controlled trial to Evaluate the effectiveness and cost benefit of prescribing high dose FLuoride toothpaste in preventing and treating dEntal Caries in high-risk older adulTs (reflect trial)

**DOI:** 10.1186/s12903-019-0749-x

**Published:** 2019-05-24

**Authors:** M. Tickle, D. J. N. Ricketts, A. Duncan, L. O’Malley, P. M. Donaldson, J. E. Clarkson, M. Black, D. Boyers, M. Donaldson, R. Floate, M. M. Forrest, A. Fraser, A. M. Glenny, B. Goulao, A. McDonald, C. R. Ramsay, C. Ross, T. Walsh, H. V. Worthington, L. Young, D. L. Bonetti, J. Gouick, F. E. Mitchell, L. E. Macpherson, Y. L. Lin, I. A. Pretty, S. Birch

**Affiliations:** 1 Division of Dentistry, University of Manchester, Coupland 3 Building, Oxford Road,M13 9PL, Manchester, UK; 2 School of Dentistry, University of Dundee, Dundee, Scotland, UK; 3 Centre for Healthcare Randomised Trials (CHaRT), University of Aberdeen, Aberdeen, Scotland, UK; 4 Northern Ireland Health & Social Care Board, Belfast, Northern Ireland; 5 NHS Education for Scotland, Edinburgh, Scotland, UK; 6 Centre for the Business and Economics of Health, University of Queensland, Brisbane, Australia; 7 Health Economics Research Unit, University of Aberdeen, Aberdeen, UK; 8 Health Services Research Unit, University of Aberdeen, Aberdeen, UK

**Keywords:** Caries, High-concentration, Fluoride, Toothpaste, Older, Adults, General dental practice

## Abstract

**Background:**

Dental caries in the expanding elderly, predominantly-dentate population is an emerging public health concern. Elderly individuals with heavily restored dentitions represent a clinical challenge and significant financial burden for healthcare systems, especially when their physical and cognitive abilities are in decline. Prescription of higher concentration fluoride toothpaste to prevent caries in older populations is expanding in the UK, significantly increasing costs for the National Health Services (NHS) but the effectiveness and cost benefit of this intervention are uncertain. The Reflect trial will evaluate the effectiveness and cost benefit of General Dental Practitioner (GDP) prescribing of 5000 ppm fluoride toothpaste and usual care compared to usual care alone in individuals 50 years and over with high-risk of caries.

**Methods/design:**

A pragmatic, open-label, randomised controlled trial involving adults aged 50 years and above attending NHS dental practices identified by their dentist as having high risk of dental caries. Participants will be randomised to prescription of 5000 ppm fluoride toothpaste (frequency, amount and duration decided by GDP) and usual care only. 1200 participants will be recruited from approximately 60 dental practices in England, Scotland and Northern Ireland and followed up for 3 years. The primary outcome will be the proportion of participants receiving any dental treatment due to caries. Secondary outcomes will include coronal and root caries increments measured by independent, blinded examiners, patient reported quality of life measures, and economic outcomes; NHS and patient perspective costs, willingness to pay, net benefit (analysed over the trial follow-up period and modelled lifetime horizon). A parallel qualitative study will investigate GDPs’ practises of and beliefs about prescribing the toothpaste and patients’ beliefs and experiences of the toothpaste and perceived impacts on their oral health-related behaviours.

**Discussion:**

The Reflect trial will provide valuable information to patients, policy makers and clinicians on the costs and benefits of an expensive, but evidence-deficient caries prevention intervention delivered to older adults in general dental practice.

**Trial registration:**

ISRCTN: 2017-002402-13 registered 02/06/2017, first participant recruited 03/05/2018.

Ethics Reference No: 17/NE/0329/233335.

Funding Body: Health Technology Assessment funding stream of National Institute for Health Research.

Funder number: HTA project 16/23/01.

Trial Sponsor: Manchester University NHS Foundation Trust, Oxford Road, Manchester, M13 9WL.

The Trial was prospectively registered.

**Electronic supplementary material:**

The online version of this article (10.1186/s12903-019-0749-x) contains supplementary material, which is available to authorized users.

## Background

The UK has an ageing population. The number of people of State Pension Age and over is projected to increase by 32.7% from 12.4 million in mid-2014, to 16.5 million by mid-2039. Likewise the number of people aged 75 and over is projected to rise by 89.3%, to 9.9 million, over the same period [1]. Whilst an increase in life expectancy should be celebrated, attention needs to be given to the complex health needs, including oral health needs, of the growing population of older people.

Dental caries is preventable, yet it is the most common disease worldwide. In high-risk older populations it is an important public health issue, as the number of older people who retain their natural teeth is growing rapidly. In England in 2009, 6% of the adult population were edentulous, compared to 28% in 1978 [2]. Dentate older adults tend to have extensively restored teeth [2], mainly due to them having grown-up prior to the widespread use of fluoride toothpaste (introduced in the 1970’s). For these older adults, the risk of developing dental caries increases due to the presence of restorations and prostheses (bridges or dentures) that increase plaque retention, dry mouth (often as a result of polypharmacy), exposed root surfaces and a cariogenic diet. The oral health of these individuals will decline as their physical and cognitive abilities deteriorate, resulting in a growing population health problem with significant financial repercussions for the NHS; so prevention of caries is important [3].

Recent National Institute for Health and Care Excellence (NICE) guidance recognised the impact poor oral health can have on older individuals’ ability to eat, speak and socialize [4]. This guidance focused on those living in care homes (either nursing or residential), however, the majority of the 11 million adults in the UK, aged 65 years or over, live in their own homes, with less than 4% (approximately 414,000) living in some form of care home [4]. It is therefore important to establish a strong evidence base for the management of the oral health needs of older people across all residential settings. In the four countries of the UK dental policy on reforming NHS dental contracts places an emphasis on General Dental Practitioners (GDPs) providing effective caries prevention [5] and they have a central role in maintaining the oral health of the older population. The significant reductions in dental caries prevalence and severity over the last 40 years have primarily been attributed to fluoride toothpaste [6]. More recently high concentration, prescription-only fluoride toothpaste has been used increasingly in the management of patients with high risk of caries [7].

The current evidence to support prescription of high concentration fluoride toothpaste (specifically 5000 ppm (ppm) toothpaste) to prevent caries is weak [8–10]. A Cochrane review of different doses of fluoride toothpaste did not include any randomised controlled trials of 5000 ppm fluoride, however the review did find a dose-response relationship between the concentration of fluoride in toothpaste and caries prevention, with greater caries prevention for higher doses of fluoride [10]. This review is currently being updated to include adults: no trials on the prevention of coronal caries through the use of 5000 ppm toothpaste have been identified. A more recent review of high fluoride concentration toothpastes included four studies (randomised and non-randomised) with 5000 ppm fluoride toothpaste [11]. None of these studies meet the inclusion criteria for the Cochrane review and all have significant design and methodological limitations. However, despite the weak evidence base, GDPs in England are advised by national guidance published by Public Health England to prescribe high concentration 2800/5000 ppm fluoride toothpaste for older adults with active caries or those at risk of developing caries [12]. The prescription of this technology is expanding; in England in 2014 1.3 million items were prescribed at a cost of £17 million [13], a 12.2% increase from the previous year and similar increases are evident in Scotland [14]. We have little understanding of how this growing NHS investment benefits patients, or if it can play a useful role in tackling an emerging public health problem. There is a need for clear, evidence-based guidance on the prescribing of high concentration fluoride toothpaste in older adults for caries prevention.

## Methods/design

### Trial aims and objectives

#### Aim

To evaluate the effectiveness and cost benefit of GDP prescribing of 5000 ppm fluoride toothpaste and usual care compared to with usual care alone in individuals 50 years and over with high-risk of caries.

Primary objective

- To compare the effect of prescribing 5000 ppm fluoride toothpaste and usual care with usual care alone on treatment for caries, including coronal/root restorations, endodontics or extractions
- To compare the costs and benefits, within a net benefit framework of prescribing 5000 ppm fluoride toothpaste with usual care

Secondary objectives will evaluate the effect of prescribing 5000 ppm fluoride toothpaste on caries (mean Decayed Missing Filled Surfaces [DMFS]) increment, progression of early caries lesions, bleeding on probing, quality of life (generic and condition specific), costs to the NHS and to individuals and society, oral health behaviour and episodes of pain. In addition, we will explore the attitudes of clinicians and patients to the prescribing and use of high fluoride toothpaste.

### Study design

A two arm, parallel group, pragmatic, open label Randomised Controlled Trial (RCT) comparing the clinical effectiveness and cost benefit of GDP prescribed high concentration fluoride toothpaste and usual care compared to usual care alone in preventing and treating dental caries in older patients. The Medicines and Healthcare products Regulatory Agency (MHRA) has ruled that the trial should be categorised as a Type A trial, as described in Risk proportionate approaches in clinical trials [15].

**Fig. 1:**
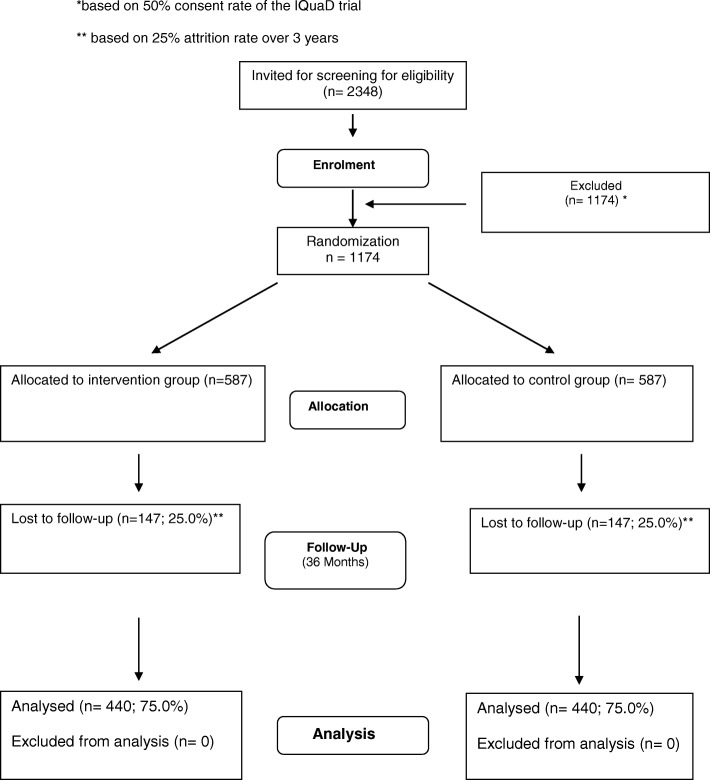
Trial Flow Diagram

A flow diagram for the trial is provided in Fig. 1. Follow up will last 3 years and will be conducted in multiple sites (approximately 60 General Dental Practices) located in England, Scotland and Northern Ireland.

In parallel with the trial we will undertake a qualitative project to understand GDPs’ practises and beliefs about prescribing 5000 ppm fluoride toothpaste and patients’ beliefs and experience of being prescribed 5000 ppm fluoride toothpaste and perceived impacts on their oral health related behaviours. Furthermore, this element will also provide GDPs’ and patients’ feedback concerning recruitment to inform our recruitment strategy. The qualitative protocol is included as Additional file 1: Appendix 1.

## Ethical considerations

Favourable ethical opinion for the REFLECT study was confirmed by the North East - Newcastle & North Tyneside 1 Research Ethics Committee on 17th November 2017 (REC reference number 17/NE/0329). The MHRA had no objection to the trial being classified as Type A under the notification scheme for risk-adapted Clinical Trials of Investigational Medicinal Products. The trial will be conducted according to the principles of Good Clinical Practice provided by Research Governance Guidelines [16]. Annual progress reports, notification of End of Trial, and a final report at the conclusion of the trial will be submitted to REC and to the MHRA within the timelines defined in the regulations. An independent Trial Steering Group supported by an independent Data Monitoring Group will oversee the conduct of the trial.

Participants are required to provide written informed consent to participate in the study, the consent form for the trial is included as Additional file 2: Appendix 2. Potential participants will receive a letter of invitation and a trial information sheet detailing the process and procedures of the trial and the nature of the intervention to be tested. Dentists will take informed consent using an approved consent form within the practices and will verbally reiterate the information contained in the information sheet and answer any questions that patients may have about the trial as part of the informed consent process. All participants will be given a £25 gift voucher at the start of the trial and another £25 voucher at the end in acknowledgment of the time that participation in the trial will entail and to thank them for their support. Consent from participants for direct access to data will also be obtained. The patients’ confidentiality will be maintained and will not be made publicly available to the extent permitted by the applicable laws and regulations.

## Study participants

Participants will be identified in multiple sites (approximately 60 General Dental Practices) located in England, Scotland and Northern Ireland. Participants will be NHS dental patients, 50 years of age or older who are considered by their dentist to be at high risk of developing caries. Our decision to use the lower limit of 50 years of age was based on: in the UK high concentration fluoride toothpaste is currently being prescribed to individuals between 50 and 60 years [17]; individuals 50 years and over have grown up in the absence of widespread use of fluoride toothpaste and consequently many individuals in this age group have heavily restored dentitions, increasing their caries risk [2]; the 2009 Adult Dental Health Survey suggests that those individuals with complex dental needs are most prevalent in late middle age [3]. Including a broad age range in the trial provides an opportunity to assess the impact of prescribing on different age groups in this older population.

High-risk individuals will be defined by: a diagnosis of active caries (into dentine) in the last 12 months which may/may not have been treated, or any root caries; and/or other risk factors as determined by their GDP. Participants can be living in any residential setting but must receive their dental care at a general dental practice (primary care). Participants will be assessed for inclusion by their GDP according to the trial inclusion and exclusion criteria.

***Inclusion criteria****:* People who

- are aged 50 years or older
- with a diagnosis of active coronal caries (into dentine) in the last 12 months which may\may not have been treated, or any root caries; and\or other risk factors as determined by their GDP.
- receive their dental care in part or fully as an NHS patient
- are living in any residential setting, and
- for whom their GDP decides prescription of high concentration fluoride toothpaste is appropriate for the patient

***Exclusion criteria:*** People who:

- are currently prescribed (by GDP or GP) high concentration fluoride toothpaste (for GDPs prescription must have been issued at last examination visit)
- hypersensitivity for Sodium Fluoride and\or other ingredients used in 5000 ppm toothpaste
- are living in the same household as someone already recruited to Reflect, or someone who is routinely using a high concentration fluoride toothpaste
- are unable to provide informed consent

## Trial intervention

The trial intervention is prescription of 5000 ppm fluoride toothpaste, used as prescribed by the participant’s GDP and informed by national guidance [12]. The intervention is designed to reflect current dental practise and therefore the frequency of prescription, the number of tubes of toothpaste per prescription and the duration of the regimen will be determined by the patient’s GDPs after assessing each patient’s risk. We have restricted the intervention to 5000 ppm fluoride toothpaste, as NHS prescribing data shows that 5000 ppm is more commonly prescribed than the 2800 ppm alternative [17].

Current guidance recommends that for those adults with obvious *active* coronal or root caries dentists should prescribe high fluoride toothpaste [12]. Our inclusion criteria are less restrictive to allow for prescription based not just on caries activity, but also based on other risk factors, as determined by the GDP. This approach is based on the hypothesis that 5000 ppm fluoride toothpaste can prevent caries in those patients deemed to be high risk by their dentist, even though they don’t present with ‘obvious’ caries. For true primary prevention, the identification of risk factors in advance of disease is indicated.

The Investigational Medicinal Product (5000 ppm fluoride toothpaste) prescribed will not be specified by manufacturer or individual product. A clinical trials pharmacy will not be used to dispense the drug. The drug will be used as prescribed by the participant’s GDP and as indicated by the British National Formulary. Participants will be expected to collect their prescriptions at a local community pharmacy as per usual practice. GDPs will provide instructions to each participant based on their assessment of risk and how they prescribe the drug.

Compliance will depend on both GDPs’ and participants’ behaviour, the trial will not seek to ensure a minimal level of compliance. Compliance will be assessed to determine its impact on trial outcomes by recording GDPs’ self-reported prescribing behaviour frequency and duration of prescriptions and the number of tubes prescribed, accessing national datasets to identify if participants’ re-deem prescriptions or receive prescriptions from other sources and via participants’ (both test and control groups) self-reported use of toothpaste.

The comparator will be usual care; usually advice given by the participant’s GDP to buy ‘standard’, off-the-shelf 1350-1500 ppm fluoride toothpaste and use as advised by their dentist informed by national guidance [12]. Other recommendations in the national guidance [12] for self-administered (mouthwash) or professionally-applied (varnish) fluoride delivery methods could have an impact on the trial. We will take a pragmatic approach and leave the decision to use other fluoride delivery systems to the patients and their dentist, but the use of these interventions will be recorded.

## Outcome measures

### Primary outcome

The proportion of participants receiving any dental treatment due to caries; including restorations, endodontics or extraction. Any dental treatment provided and the reasons for providing treatment (caries, tooth wear or other e.g. trauma) will be recorded. The primary outcome (treatment directly due to caries) will be extracted from the Clinical Report Form (CRF).

### Secondary outcomes (clinically assessed)

The clinically assessed secondary outcomes will be measured in Scottish practices only; Additional file 3: Appendix 3 provides a sub-protocol for this more intensive assessment of participants

- Coronal caries increment, including dentist replacement restorations for caries, at tooth surface (DMFS) level by independent, trained and calibrated, clinical examiners at baseline and 3 year (+/− 3 months) follow-up. We will use the ICDAS method [18] to assess caries as it provides flexibility to analyse and present caries data at different diagnostic thresholds.
- Root caries increment, including dentist replacement restorations for caries, at tooth surface (DMFRS) level by independent, clinical examiners at baseline and 3 year (+/− 3 months) follow-up.
- Early caries lesion progression data, using ICDAS.
- Bleeding on probing (BoP) will also be recorded by the independent clinical examiners. BoP provides evidence of long term optimal brushing rather than transitory measures such as visible plaque scores [19].

### Secondary outcomes (patient reported)

- Oral health status using OHIP14, a measure of oral health-related Quality of Life (QoL), collected at baseline and annual follow up through patient administered questionnaires. The OHIP-14 is the most common, validated dental quality of life instrument and has been successfully used in previous HTA trials. It has been found to be sensitive to differences in oral health and is closely correlated with self-reported oral health outcomes [20]
- The EQ-5D-5 L profile measure of generic health status [21, 22], will be collected at baseline and annual follow up through patient questionnaires.
- Any episode of dental pain (and total number per participant) during the 3 year follow up period, severe enough to trigger seeking advice from a healthcare professional (dentist, GP, community pharmacist). This will be recorded at scheduled and unscheduled dental visits by patient questionnaire included in the CRF.
- Oral health behaviour, including self-reported brushing/other sources of fluoride. Evaluated at baseline and through annual questionnaires sent by mail to the home address of participants.

### Routine data

- Fulfilment of prescriptions will be measured via Business Services Authority (BSA), Business Services Organisation (BSO) and Information Services Division (ISD) datasets. Adherence to the allocated intervention (prescription of 5000 ppm) will also be explored by dentist and participant self-reporting (via CRF) and in the qualitative interviews with patients

### Economic outcomes

- Provision of NHS and privately-funded dental treatments will be collected from the CRF. In addition we will also collect information on NHS treatment completed using routinely collected NHS data held by the ISD, BSA and BSO. Treatment cost data will be collected for participants from 1 year prior to randomisation until the end of the trial.
- All remaining resource use data will be collected using questionnaires provided to participants on attendance for dental care or mailed to participants who fail to attend. These will include the use of other NHS healthcare services (e.g. GP visits, dental hospital attendances and other healthcare resource use) directly related to dental problems as well as data on non-NHS costs (e.g. time and travel costs, time off work, privately purchased care, self-purchased dental care products).
- Collection of EQ-5D-5 L will enable the calculation of the mean cost per additional QALY as a secondary health economic outcome.
- A discrete choice experiment (DCE) with an online representative sample of the UK general population (aged 50 and over) will be undertaken to elicit willingness to pay (WTP) for high fluoride toothpaste and associated patient relevant outcomes
- Long term economic evaluation with trial results extrapolated over a life-time horizon using Markov modelling methods

The methods used to collect the various outcome measures are summarised in Table 1.

**Table 1:** Summary of methods used to collect outcome measures

	Baseline	Elective and non-elective visits to the dentist over 3 year follow up^a^	36 months	Annual Questionnaire	National Database
Clinical Status (full dental chart)	○	○	○		
Treatment details Check ups, restorations, endodontics, extractions (reason for treatment in CRF and all NHS treatments in national datasets) Prescription of toothpaste (date and amount prescribed) Relevant medical history and medication	○	○			X
Healthcare costs GDP completed CRF: payment mechanism (NHS/private) and charges levied Dental treatment received in other settings (patient element of CRF) NHS costs from national data sets Non NHS treatment costs in patient element of CRF)	○	○	○		X
Detailed independent clinical assessment ICDAS, BoP (Scotland only)	○		○		
EQ-5D-5 L	○			●	
OHIP-14	○			●	
Pain - seeking professional care because of pain	○	○			
Oral health behaviour	○			●	
Redemption of toothpaste prescriptions	○	○	○		X
Adverse reactions		○			

^a^dentists should invite participants to attend practice at least once a year

○ Dental Practice – CRF (includes both GDP and participant completed elements)

● Postal Questionnaire

X NHS centralised dental databases

## Follow up of participants

Participants will be followed up for a 3-year period. Participants will remain in the trial unless they choose to withdraw consent or if they are unable to continue for clinical reasons. All changes in status with the exception of complete withdrawal of consent will mean the participant is still followed up for all trial outcomes wherever possible. Participants randomised to 5000 ppm fluoride toothpaste, who for whatever reason, stop using the trial medication or participants randomised to routine care but who purchase or otherwise receive high fluoride toothpaste will not be considered to have withdrawn from the trial.

The Sponsor and CI have undertaken an initial risk assessment, which will be periodically reviewed and if necessary updated as the trial progresses. The Sponsor has concluded that this is a low risk trial, as the drug is being prescribed by GDPs as per its licensed indications. It is topically administered and the known side effects are rare and minor in nature [23].

Any Serious Adverse Reactions (SARs) will be recorded from the time a participant consents to join the study until the end of their follow up. The local investigator (GDP) will record in the CRF all directly observed Adverse Reactions (ARs) and all ARs spontaneously reported by participants that have a possible causal link to the IMP. In addition, each trial participant will be asked to complete a questionnaire at each attendance at their dental practice, and one sent annually to their home, which collects information on potential ARs and SARs.

The Sponsor and CI will ensure, through the independent Trial Steering Committee, that adequate systems are in place for monitoring the quality of the trial (compliance with appropriate governance) and appropriate expedited and routine reports, to a level appropriate to the risk assessment of the trial.

## Randomisation

Randomisation will be at patient level. Eligible and consenting participants will be randomised to intervention and control groups using a web-based application, hosted by the Clinical Trials Unit (CTU) CHaRT (Centre for Healthcare and Randomised Trials). The Principal Investigator (PI), or individual at site with delegated authority, will access the web-based system to randomise participants. The randomisation algorithm will use recruitment site, residential setting (own home/care home), exemption from dental treatment charges (yes/no) and age (50–65 years/over 65 years) as minimisation covariates to allocate treatment to intervention and control groups in a 1:1 ratio. A random element will be incorporated into the randomisation algorithm. Participants will be informed of their allocated treatment group following randomisation. The trial statistician and the study team will be blinded to the allocation during all analyses by use of a code to identify the two groups. The key to the code will be held by the CTU.

Blinding of outcome assessment using the primary outcome will not be possible, as the participant’s dentist will collect primary outcome data. A more detailed clinical examination undertaken by independent (external to the trial dental practices) examiners will be used to repeat primary outcome measures and collect secondary outcomes (coronal and root caries increments and bleeding on probing). This more detailed clinical examination will take place in Scottish practices only and the independent examiners will be blind to the allocation. Source data verification will be undertaken according to the monitoring plan by:

1. Comparing the primary outcome measures with outcomes recorded in the clinical examination (Scotland only)
2. Comparing primary outcome measure in the CRF treatment provided for caries (coronal and root surfaces) including restoration, extraction and endodontics with centralised NHS treatment claims data for individual patients.
3. Source data verification audits in annual visits of each practice.

## Sample size considerations

The sample size calculation is based on a meaningful absolute target difference of 10% (75% vs 65%) in the primary outcome measure. This difference is considered to be both a realistic and important difference from discussion with dentists, PPI groups and from published estimates [6, 7]. The value for the comparator group (75% of individuals allocated to standard care who have restoration(s) or extraction(s) due to caries during the 36 months of follow up) is based on published data and Scottish treatment data [24]. For the proposed target difference, a two-sided 5% significance level, and 90% power, 440 participants (880 in total) will be required to provide data for the primary outcome at 36 months. Based on our previous and current HTA trials, we are assuming 25% attrition, and so 587 participants per group are required (1174 in total) in approximately 60 practices (each practice recruiting an average of 20 participants). Based on an estimated consent rate of 50% [data from IQuad [25]], 2348 eligible patients will be invited to participate.

An important secondary outcome within our proposed trial is caries increment, measured using the number of Decayed Missing and Filled tooth Surfaces (DMFS). Using the mean number of Decayed Missing and Filled tooth Surfaces (DMFS), the caries increments for an older population in the published literature vary, but there seems to be consensus around one surface per year [26]. Given the fact that the standard deviations approximate the means in terms of caries increment, a reduction in caries increment from 3 to 2 surfaces with the intervention would produce a ~ 30% reduction in caries increment with the intervention over 3 years. The numbers needed to adequately power this secondary outcome are relatively small compared with the primary outcome measure:

For secondary caries outcomes (Scotland only), group sample sizes of 200 and 200 achieve 97.5% power to reject the null hypothesis of equal means when the population mean difference DMFS increment is μ1 - μ2 = 2–3 = − 1.0 with standard deviations of 2 for group 1 (intervention) and 3 for group 2 (control), and with a significance level (alpha) of 0.05 using a two-sided two-sample t-test allowing for unequal variances. Assuming 25% attrition, 267 participants per group are required (534) in total in 28 practices. Based on an estimated consent rate of 50% 1068 eligible patients will be invited to participate.

## Statistical analysis

Statistical analysis will be conducted according to a detailed statistical analyses plan (SAP) which will include, but not be limited to the investigational plan and study design, listing of outcomes and final analysis including effectiveness evaluations. The SAP will set out the summary measures to be reported; methods of analysis, plans for handling missing data, non-compliance and withdrawals, the timing and frequency of analyses, and use of intention to treat analysis.

Demographic (age, sex) and baseline characteristics (DMFT, DMFS, concomitant illness / treatment, exempt\not exempt from NHS dental charges) will be summarised and displayed in tables for all randomised patients. In addition, mean NHS treatment costs in the 12 months prior to recruitment will be included in the baseline characteristics tables. Frequency counts and percentages will be used to present categorical data. Number of patients, mean, mode, median, SD, minimum, maximum and IQR will be used, as appropriate, to present continuous data.

The primary outcome measure will be analysed using a generalised linear model with the appropriate link function adjusting for the minimisation variables (recruitment site, residential setting, exemption (including partial exemption) from dental treatment charges and age band). Secondary outcomes will be analysed using generalised linear models with adjustment for minimisation and baseline variables when available. Statistical significance will be at the 2-sided 5% level with corresponding confidence intervals derived. Subgroup analyses on the primary outcome will explore the possible modification of treatment effect by clinically important factors; gender, age and NHS dental charges exemption status. This will be done by including treatment-by-factor interactions in the model and they will be classified as exploratory analyses. All analyses will initially be performed on an intention to treat basis, although we will consider additional analysis groups such as per-protocol for investigation of adverse events.

Outcome data will not be imputed for the primary analysis, but score data for participants who have not returned a scheduled questionnaire will be estimated using a multiple imputation approach to make use of partial outcome data. Sensitivity analyses will be conducted to assess the robustness of the treatment effect estimate to these approaches. Missing items on the health-related outcome measures will be treated as per the instructions for that particular measure. There are no planned interim outcome analyses; all analyses will occur following completion of trial follow up. Interim analyses will be performed only if requested by the Independent Data Monitoring Committee

## Economic evaluation

A full economic evaluation will be conducted as part of this study:

1. At 3 years of follow up, alongside the primary RCT outcome measure, and
2. Based on an extrapolation of trial outcomes over a patient’s life-time, using an appropriate Markov decision analysis model to explore longer term cost-effectiveness.

The primary economic evaluation will be in the form of a cost-benefit analysis, reporting net benefit (Willingness To Pay (WTP) - cost), with WTP elicited using a Discrete Choice Experiment (DCE). We have chosen a cost-benefit analysis (CBA), as opposed to a traditional analysis of cost per generic EQ-5D QALY as the primary economic outcome measure because of concerns that generic QALYs are not sufficiently sensitive to capture the processes and outcomes of dental treatments. The CBA is therefore the outcome of most interest in terms of investigating value for money (efficiency) of prescription of high concentration fluoride toothpaste. In addition to the primary health economic analysis, two further analyses will be undertaken as secondary economic outcomes to inform various stakeholders:

- NHS decision makers, such as NICE may be interested in the cost of achieving gains in quality adjusted life years (QALYs) and therefore recommend the conduct of a cost per QALY analysis for technology appraisal. In order to comply with these recommendations, we will include the generic EQ-5D-5L health profile measure at baseline and each follow up time point in the trial. This will enable the calculation and presentation of the mean cost per additional QALY as a secondary health economic outcome.
- Dental practitioners may be interested in the cost of achieving various specific clinical outcomes. We will therefore complete a cost-effectiveness analysis, based on the primary clinical outcome measure for the trial, reporting cost per episode of dental treatment avoided (i.e. the cost per filling or extraction avoided). A further secondary analysis will present cost per DMFS avoided.

### Estimation of costs

Costs will be estimated from both an NHS and patient perspective. NHS costs of providing the high fluoride toothpaste intervention will be based on the costs of dispensed prescriptions verified by BSA, BSO & ISD in England, Northern Ireland and Scotland respectively. Methods for the collection of resource use data for cost estimation over the trial follow up are summarised in Table 1. All resource use data will be costed at the patient level, using region specific tariffs, and aggregate costs applied across these three settings, with sensitivity analyses presenting data for each setting separately.

Data on costs for each area of service use will be summed to provide a mean cost per patient participant (from both an NHS and patient perspective). Incremental costs per patient for high dose fluoride toothpaste vs usual care will be estimated using generalised linear models with appropriate distributions for cost data and adjustment for baseline covariates, such as gender and age. The costing analysis will include a statement on budget impact of alternative policy approaches.

### Cost-Benefit Analysis – willingness to pay (WTP)

Whilst health based outcomes are of importance for funders of dental care, there is a growing interest in wider measures of value, which go beyond traditional QALY approaches and offer a more holistic and sensitive measure of value. It is crucially important for adherence, and hence real-world cost-effectiveness, that any public health intervention is not only valued in terms of health outcomes within a clinical trial, but also has wider generalisability to the consumers of the intervention. We will therefore conduct a cost-benefit analysis (CBA) reporting benefits in terms of WTP to obtain a more holistic measure of value. WTP will be obtained from a discrete choice experiment (DCE) [27], conducted with a nationally representative sample of the general population (aged 50 and over), to explicitly value the provision of high fluoride toothpaste, together with a range of plausible outcomes from the trial (e.g. avoidance of caries progression). The DCE will explicitly value the high fluoride toothpaste intervention, together with associated health outcomes and other important attributes.

The DCE will include a cost attribute. By including this attribute, the willingness to pay (WTP) for a change in the level of any other attribute will be estimated. Estimates of WTP derived from the DCE will be combined with the intervention (provided or not) and clinical outcome data from the trial to report net benefit [mean WTP – mean cost [28]] for high fluoride toothpaste compared to standard care. If benefits are greater than the costs, then high fluoride toothpaste would be deemed an efficient use of resources.

Results will be presented on a cost-benefit plane, illustrating the probability that the intervention is associated with positive or negative net benefit. A comprehensive set of sensitivity analyses will be undertaken to explore uncertainty in our conclusions. These will include assumptions surrounding missing data, the estimation of costs and the effect of different payment / co-payment structures on the cost-benefit results.

### Decision modelling

The “within trial” economic analyses will assess and report on the costs and outcomes of high fluoride vs. standard treatment up to 3 years post-randomisation. However, the true economic value of an intervention depends on the long-term implications of that intervention. We will develop a de novo Markov decision analysis model, to extrapolate the trial outcomes over a longer, life-time horizon [29]. Results will be reported using a cost-effectiveness framework and estimates of WTP from the DCE will be used to explore results using a similar cost-benefit framework to that used for the primary trial based economic analysis. The final model structure and health state definition (e.g. caries progression, new caries, and tooth loss) will be developed in conjunction with dental experts. National cohort datasets, such as the adult dental health survey, and other longitudinal studies will be used as a source of baseline transition probabilities. Sensitivity analysis will use the data from the control (standard care) arm of the trial. Where possible, survival analysis methods will be used to assess the time to transition between health states in each of the study arms, with survival curves fitted over an extended time frame (patient’s life time). Data from cohort studies and literature reviews will be used where necessary to supplement extrapolation models to determine long run caries progression.

Cost data for health states beyond trial follow up will be sourced from the trial data and routine data sources (ISD/BSA/BSO) for appropriate treatment in the respective model health states. To inform a life-time cost-benefit analysis, estimates of WTP will be sourced directly from the DCE conducted alongside the trial for specific health states (e.g. WTP to avoid caries progression, new caries or tooth loss). The model will be developed at an early stage in the study to ensure all relevant data to populate the model are collected within the trial. All modelling assumptions will be extensively tested using sensitivity analyses and the model will be fully probabilistic. Key gaps in the evidence base will be identified and their potential impact on cost-effectiveness explored. Results will be presented using standard economic evaluation approaches to illustrate uncertainty. Threshold analyses will be conducted to indicate the values required for key model parameters to change economic evaluation results. Patients will be consented to obtain longer-term data linkage to routine records, which can then be used for future validation of the extrapolation assumptions.

## Research governance, data protection and sponsorship

The trial will be run under the auspices of CHaRT based at HSRU, University of Aberdeen. This will aid compliance with Research Governance, and provide centralised trial administration, database support and economic and statistical analyses. CHaRT is a registered Clinical Trials Unit with particular expertise in running multicentre RCTs of complex and surgical interventions. The Sponsor and CI will ensure, through the TSC, that adequate systems are in place for monitoring the quality of the trial (compliance with appropriate governance) and appropriate expedited and routine reports, to a level appropriate to the risk assessment of the trial.

The Trial Co-ordinating Office (TCOD) based in the Dundee Dental School and Hospital, University of Dundee will provide day-to-day support to the GDPs and outcome assessors/research nurses. The Trial Office Teams at TCOD and CHaRT will meet formally approximately monthly during the course of the trial to ensure smooth running and trouble-shooting.

Data collected during the course of the research will be kept strictly confidential and accessed only by members of the trial team. Participants’ details will be stored on a secure database under the data protection guidelines and regular checks and monitoring are in place to ensure compliance. Data will be archived to a secure data storage facility. The CTU senior IT manager (in collaboration with the Chief Investigator) will manage access rights to the data set. Participants will be allocated an individual specific trial number and their details will be anonymised on the secure database. We anticipate that anonymised trial data may be shared with other researchers to enable international prospective meta-analyses.

Manchester University NHS Foundation Trust is the sponsor for the trial. An independent risk assessment was carried out by the Sponsor prior to commencement of the trial and will be reviewed and, if necessary, updated annually for the duration of follow up. The trial will be monitored to ensure that the study is being conducted as per protocol, adhering to Research Governance, and the appropriate regulations. The approach to, and extent of, monitoring will be specified in a trial monitoring plan which will be determined by the risk assessment of the study and updated if necessary following the outcomes of the annual risk assessment review.

The Sponsor will provide indemnity for non-negligent harm. All dentists and dental care professionals working on the trial will be registered with the General Dental Council and have appropriate indemnity arrangements in place.

### Data-handling, record keeping and archiving

Clinical data will be entered into the trial database by the TCOD together with data from questionnaires completed at clinic. Questionnaires returned by post to the trial office will be entered there. Staff in the trial office will work closely with practices to ensure that the data are as complete and accurate as possible. Extensive range and consistency checks will further enhance the quality of the data. The Sponsor is responsible for ensuring that trial data is archived appropriately. Essential data shall be retained for a period of at least 15 years following close of trial.

### Satellite studies

It is recognised, that the value of the trial may be enhanced by smaller ancillary studies of specific aspects. Plans for these will be discussed in advanced with the Project Management Group. REC approval will be sought for any new proposal, if appropriate.

## Dissemination

This trial will produce new knowledge which will be valuable to key stakeholder groups: patients, policy makers, dental healthcare providers and commercial organisations both in the UK and internationally. We intend to maintain interest in the trial by publication of newsletters at intervals for staff and collaborators. Once the main report has been published, a lay summary of the findings will be sent in a final Newsletter to all involved in the trial.

We will develop our dissemination strategy as the trial progresses with our PPI group and engagement with stakeholder groups. The key stakeholders will be:

1. Policy makers, guideline producers and commissioners: the four Chief Dental Officers (CDOs), NICE, SIGN, SDCEP and Public Health England. At the end of the trial we intend to hold a workshop for policy makers and produce summary briefing documents of our findings. The trial team will liaise with the editorial team from the Cochrane Oral Health Group to ensure the timely update of the relevant Cochrane reviews. We will engage with international bodies and organisations such as, WHO, US Centre for Disease Control, FDI.
2. Clinicians: we will speak at national clinical conferences, relevant dental specialist society meetings and produce clinical summary papers in popular journals read by clinicians.
3. Patients and the public: we will issue press releases to the popular media and post lay summaries of the outcomes on our web sites and engage with the public through social media.
4. Academic community: the principal output will be a monograph published in accordance with NIHR guidelines on project outputs [30] in the funder’ journal. In addition, we will aim to publish our findings in peer-reviewed, high impact, internationally-leading journals. We will present our findings at relevant national and international oral health conferences such as IADR.
5. Industry and commerce: We will provide reports for major toothpaste manufacturers.

## Discussion

The Reflect Trial is an NIHR HTA funded trial that is being undertaken across England, Northern Ireland and Scotland. It is a pragmatic, multi-centre, open-label randomised trial designed to determine whether or not the substantial and rapidly increasing costs of prescribing high fluoride concentration toothpaste benefits patients and represents a wise investment for the UK National Health Service. The trial is pragmatic in nature, designed as far as possible to mirror ‘real life’. This pragmatic approach is reflected in:

- the population: involved in the trial, high caries risk older adults, have been identified by national guidance in England as a key target group for prescribing high concentration fluoride toothpaste [12]
- the intervention: prescription of high concentration fluoride toothpaste. 5000 ppm fluoride toothpaste was chosen, as this higher concentration is likely to produce the largest effect size (compared to 2800 ppm fluoride toothpaste). The intervention tests the prescription of the drug by GDPs rather than the drug being directly provided to participants. Therefore decisions taken by dentists on the amount and frequency of prescribing the drug and the compliance of participants in using the toothpaste need to be measured and considered in the analysis and interpretation of results.
- the primary outcome measure: receipt of treatment as a result of caries was determined by patient and public involvement as a key concern of patients. More traditional measures of caries are included as secondary outcome measures
- the comparator: the intervention will be compared to ‘usual practice’ as determined by each participant’s dentist.

Importantly the setting for the trial is primary care dental practices, where the overwhelming majority of patients receive their dental care. Participants will be recruited in approximately 60 practices across the three countries, providing a varied range of practices operating in different geographical environments and socio-economics circumstances and different remuneration systems. This broad range of practices should ensure the results of this trial are widely applicable. The evidence base for high concentration fluoride toothpaste is not strong both in terms of both efficacy and for how well this health technology works in day-to-day general dental practice. The results of this pragmatic trial should complement the findings of efficacy trials to provide high quality evidence on the costs and benefits of high concentration fluoride toothpaste to support the decisions of dental practitioners, patients and policy makers on its use.

The team responsible for the design and delivery of the trial is multidisciplinary drawn from a strong collaboration between the Universities of Aberdeen, Dundee and Manchester plus colleagues working in the NHS and local research networks. The trial is a Clinical Trial of a Investigative Medicinal Product and is overseen by the trial sponsor Manchester University Foundation Trust, supported by an independent Trial Steering Group and Data Management Committee and Patient and Public Involvement Group. This will ensure that the trial is conducted to the highest standards. We expect the Reflect Trial to make an important national and international contribution to understanding the role that high concentration fluoride toothpaste can play in the management of high caries risk in older adults attending primary dental care.

## Trial status

Currently recruiting. Participant recruitment began in May 2018 and is expected to finish recruiting in August 2019. The first participant was randomised on 03/05/2018. Current approved protocol: Version 1.2, 16/01/2018.

## Additional files

Additional file 1: **Appendix 1.** Qualitative study protocol. (DOCX 16 kb)

Additional file 2: **Appendix 2.** Consent form. (DOCX 237 kb)

Additional file 3: **Appendix 3.** Scottish sub-protocol. (DOCX 13 kb)

Additional file 4: **Appendix 4.** SPIRIT checklist. (DOCX 43 kb)

## Abbreviations

AE: Adverse Event
AR: Adverse Reaction
AUC: Area under the curve
BID: Twice a day
BNF: British National Formulary
BoP: Bleeding on probing
BSA: Business Services Authority
BSO: Business Services Organisation
CBA: Cost-Benefit Analysis
CEAC: Cost-effectiveness Acceptability Curve
CHaRT: Centre for Healthcare Randomised Trials
CI: Chief Investigator
CONSORT: Consolidated Standards of Reporting Trials
CRF: Clinical Report Form
CTA: Clinical Trial Application
CTIMP: Clinical Trial of an Investigational Medicinal Product
CTU: Clinical Trial Unit
DCE: Discrete Choice Experiment
DMFRS: Decayed Missing Filled Root Surfaces
DMFS: Decayed Missing Filled Surfaces
DMFT: Decayed Missing and Filled Teeth
DSUR: Development Safety Update Report
EQ-5D: EuroQol Group’s 5 dimension health status questionnaire
EudraCT: European Union Drug Regulating Authorities Clinical Trials
GCP: Good Clinical Practice
GDP: General Dental Practitioner
GP: General Practitioner
HRQoL: Health Related Quality of Life
HSRU: Health Services Research Unit
HTA: Health Technology Assessment
IB: Investigator Brochure
ICDAS: International Caries Detection and Assessment System
IDMC: Data Monitoring Committee
IMP: Investigational Medicinal Product
IQR: Inter Quartile Range
ISD: Information Statistics Division
ISF: Investigator Site File
ISRCTN: International Standard Randomised Controlled Trial Number
IVR: Interactive Voice Response (randomisation)
MHRA: Medicines and Healthcare products Regulatory Agency
MRC: Medical Research Council
NCT: National Clinical Trial
NHS: National Health Service
NHSG: National Health Service Grampian
NICE: National Institute for Health and Care Excellence
NIHR: National Institute Health Research
NRES: National Research Ethics Service
OD: Once a day
OHIP: Oral Health Impact Profile
PI: Principal Investigator
PIL: Patient Information Leaflet
PMG: Project Management Group
PPI: Patient Public Involvement
PQ: Participant Questionnaire
QALY: Quality Adjusted Life Year
QID: Four times a day
R&D: Research and Development
RCT: Randomised Controlled Trial
REC: Research Ethics Committee
SAE: Serious Adverse Event
SAP: Statistical Analysis Plan
SAR: Serious Adverse Reaction
SD: Standard Deviation
SmPC: Summary of Product Characteristics
SOP: Standard Operating Procedure
SUSAR: Suspected Unexpected Serious Adverse Reaction
TCOD: Trial Coordinating Office Dundee
TMF: Trial Master File
TSC: Trial Steering Committee
UAR: Unexpected Adverse Reaction
UK: United Kingdom
UKCRC: United Kingdom Clinical Research Collaboration
UoA: University of Aberdeen
WTP: Willingness to pay
