# Supplementary material for: Protocol for a Randomised controlled trial to Evaluate the effectiveness and cost benefit of prescribing high dose FLuoride toothpaste in preventing and treating dEntal Caries in high-risk older adulTs (reflect trial)
Source: BMC Oral Health. 2019 May 24;19:88. doi: 10.1186/s12903-019-0749-x (PMC6534863; doi:10.1186/s12903-019-0749-x)
Supplement: Supplementary file 1 — Appendix 1. Qualitative study protocol. (DOCX 16 kb) [file 12903_2019_749_MOESM1_ESM.docx]

**Additional file 1: Appendix 1 Qualitative element**

Objectives

The qualitative investigation will be based on semi-structured interviews with patients aged 50 years of age or older and GDPs. A semi-structured interview approach is selected due to its flexible and interactive nature, which is useful to understand decision-making process and the shaping of individual beliefs. The objectives are to investigate GDPs’ practices of and beliefs about prescribing high dose fluoride toothpaste and patients’ beliefs and experience of being prescribed high dose fluoride toothpaste and perceived impacts on their oral health related behaviours. Furthermore, the qualitative element will also ask for GDPs’ and patients’ feedback concerning the design of the trial for subsequent evaluation of the project. The interviews with GDPs will explore when and how they decide to prescribe 5000ppm toothpaste and their perceived benefits and drawbacks of it. Furthermore, the ways in which their beliefs are shaped will also be investigated to understand how GDPs come to hold certain beliefs and how these beliefs affect their clinical practice. Patients’ views about being prescribed high dose fluoride toothpaste will also be explored, particularly the ways in which the prescription affects their everyday life, routines and beliefs. GDPs and patients will also be consulted about their experience in taking part in the trial, including their views about the topic, the reason they agreed to participate in the study and what can be improved. This provides a real-time and continuous evaluation of the research design, which can be used to cross examine the validity and reliability of the trial.

Recruitment

NHS dental patients, 50 years of age or older, attending the GDP who are considered to be at high risk of developing caries. GDPs will be recruited from approximately 60 practices through the existing network. Consent to be interviewed from GDPs and patients will be acquired when they are recruited for the trial. Participants will be asked if they are willing to be contacted for interviews whilst consenting to the trial. A qualitative researcher will be given the contact details of participants who consented to be interviewed and arrange telephone interviews based on participants’ preference.

Data collection

Data collection will start with GDPs immediately after their consent but it will only commence four weeks after patient’s consent to the study. This is to allow time for patients in the intervention groups to get used to using high dose fluoride toothpaste so that they have sufficient experience to answer questions in the interviews. The interviews will be conversational and explorative. Although an interview topic guide will be used to remind the interviewer the topics to be covered, it is expected that the conversation will follow the flow of the discussion and probing questions will be asked to tease out in-depth information ([Bryman 2012](#_ENREF_2)). We estimate that interviews will take approximately 45-60 minutes but the length of each interview will be guided by the conversation flow. Data collection will continue until theoretical data saturation is reached. Follow-up interviews will be conducted with participants at the end of the trial to explore changes in attitudes over time and investigate GDP and patients’ experiences of the trial.

Data management and analysis

All interviews will be audio-recorded with consent from participants and transcribed verbatim. Transcripts will be imported to qualitative analysis software (NVivo) for data management and explored using thematic analysis. The researcher will read and re-read the data to familiarise herself with the emerging ideas. Codes will then be generated in a systematic fashion across the entire data set. These codes will be collated into potential themes which will then be reviewed and refined ([Braun and Clarke 2006](#_ENREF_1)).

Confidentiality and anonymity

Audio recording of interviews and transcripts will be either encrypted or password protected. These will be kept on the University of Manchester’s secure IT service. Participants will be given a codename in the data analysis where all identifiable information will be removed. Once the study is concluded, all recordings, transcripts and documents related to this project will be stored securely at the University of Manchester for 5 years following publication of the study.

Braun, V. and V. Clarke (2006). "Using thematic analysis in psychology." Qualitative Research in Psychology **3**(2): 77-101.

Bryman, A. (2012). Social research methods. Oxford, Oxford University Press.
