# Supplementary material for: Protocol for a Randomised controlled trial to Evaluate the effectiveness and cost benefit of prescribing high dose FLuoride toothpaste in preventing and treating dEntal Caries in high-risk older adulTs (reflect trial)
Source: BMC Oral Health. 2019 May 24;19:88. doi: 10.1186/s12903-019-0749-x (PMC6534863; doi:10.1186/s12903-019-0749-x)
Supplement: Supplementary file 2 — Appendix 2. Consent form. (DOCX 237 kb) [file 12903_2019_749_MOESM2_ESM.docx]

**Additional file 2: Appendix 2 Informed Consent Form**
