# Supplementary material for: Protocol for a Randomised controlled trial to Evaluate the effectiveness and cost benefit of prescribing high dose FLuoride toothpaste in preventing and treating dEntal Caries in high-risk older adulTs (reflect trial)
Source: BMC Oral Health. 2019 May 24;19:88. doi: 10.1186/s12903-019-0749-x (PMC6534863; doi:10.1186/s12903-019-0749-x)
Supplement: Supplementary file 3 — Appendix 3. Scottish sub-protocol. (DOCX 13 kb) [file 12903_2019_749_MOESM3_ESM.docx]

**Additional file 3: Appendix 3 Scottish sub-protocol**

Collection of Clinical Outcome Measures (Scotland only)

All clinical outcomes will be assessed at baseline and three years by trained examiners who are blinded to allocation. Training will be provided by an expert in caries assessment and the use of criteria in caries clinical trials.

The independent examiner will first duplicate the clinical data collection as recorded by the GDP (the condition of coronal and root surfaces).

A detailed caries measurement will be made using the validated International Caries Detection and Assessment System (ICDAS) for coronal caries. The ICDAS criteria measure both early and more advanced stages of caries. For early caries, ICDAS measures the surface changes and potential histological depth of carious lesions by relying on surface characteristics related to the optical properties of sound and demineralised enamel prior to cavitation. The primary requirement for applying the ICDAS system is the examination of clean and dry teeth aided by a ball-ended explorer that is used to remove any remaining plaque and debris and to check for surface contour, minor cavitation or sealants. All surfaces of all teeth will be examined and the caries status recorded.

Periodontal Gingival inflammation as bleeding will be measured according to the Gingival Index of Loe by running a UNC periodontal probe circumferentially around each tooth just within the gingival sulcus or pocket. After 30 seconds, bleeding will be recorded as being present or absent on the buccal and lingual surfaces.
