# Supplementary material for: Protocol for a Randomised controlled trial to Evaluate the effectiveness and cost benefit of prescribing high dose FLuoride toothpaste in preventing and treating dEntal Caries in high-risk older adulTs (reflect trial)
Source: BMC Oral Health. 2019 May 24;19:88. doi: 10.1186/s12903-019-0749-x (PMC6534863; doi:10.1186/s12903-019-0749-x)
Supplement: Supplementary file 4 — Appendix 4. SPIRIT checklist. (DOCX 43 kb) [file 12903_2019_749_MOESM4_ESM.docx]

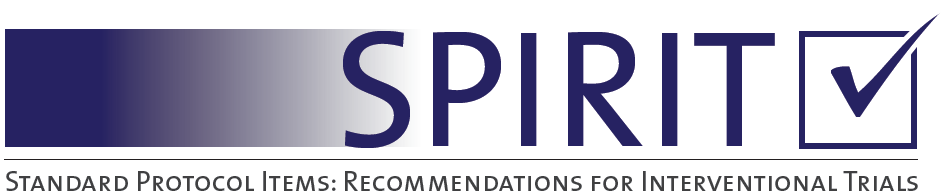
**Additional file 4 Appendix 4 SPIRIT checklist**

SPIRIT 2013 Checklist for: **Protocol for a Randomised controlled trial to Evaluate the effectiveness and cost benefit of prescribing high dose FLuoride toothpaste in preventing and treating dEntal Caries in high-risk older adulTs (Reflect trial)**

| Section/item | ItemNo | Description |
| --- | --- | --- |
| **Administrative information** | | |
| Title | 1 | Descriptive title identifying the study design, population, interventions, and, if applicable, trial acronym  **Title P1** |
| Trial registration | 2a | Trial identifier and registry name. If not yet registered, name of intended registry  **Abstract P3** |
|  | 2b | All items from the World Health Organization Trial Registration Data Set  **Abstract P3** |
| Protocol version | 3 | Date and version identifier  **P15 Trail Status** |
| Funding | 4 | Sources and types of financial, material, and other support  **P3 Abstract** |
| Roles and responsibilities | 5a | Names, affiliations, and roles of protocol contributors  **P1 Title page** |
|  | 5b | Name and contact information for the trial sponsor  **P3 Abstract** |
|  | 5c | Role of study sponsor and funders, if any, in study design; collection, management, analysis, and interpretation of data; writing of the report; and the decision to submit the report for publication, including whether they will have ultimate authority over any of these activities  **P13 Research governance, data protection and sponsorship, P15 in Discussion second paragraph and in Declarations under Ethics approval and consent to participate** |
|  | 5d | Composition, roles, and responsibilities of the coordinating centre, steering committee, endpoint adjudication committee, data management team, and other individuals or groups overseeing the trial, if applicable (see Item 21a for data monitoring committee)  **P13 under Research governance, data protection and sponsorship** |
| Introduction |  |  |
| Background and rationale | 6a | Description of research question and justification for undertaking the trial, including summary of relevant studies (published and unpublished) examining benefits and harms for each intervention  **P4 Background** |
|  | 6b | Explanation for choice of comparators  **P5 in Methods/Design under Aims and objectives, P7 paragraph 5, P14 in Discussion 4^th^ bullet point** |
| Objectives | 7 | Specific objectives or hypotheses  **P5 under Aims and objectives** |
| Trial design | 8 | Description of trial design including type of trial (eg, parallel group, crossover, factorial, single group), allocation ratio, and framework (eg, superiority, equivalence, noninferiority, exploratory)  **P5 under Study design** |
| Methods: Participants, interventions, and outcomes | | |
| Study setting | 9 | Description of study settings (eg, community clinic, academic hospital) and list of countries where data will be collected. Reference to where list of study sites can be obtained  **P6 under Study participants first paragraph** |
| Eligibility criteria | 10 | Inclusion and exclusion criteria for participants. If applicable, eligibility criteria for study centres and individuals who will perform the interventions (eg, surgeons, psychotherapists)  **P6 under Study participants** |
| Interventions | 11a | Interventions for each group with sufficient detail to allow replication, including how and when they will be administered  **P7 under Trial intervention** |
|  | 11b | Criteria for discontinuing or modifying allocated interventions for a given trial participant (eg, drug dose change in response to harms, participant request, or improving/worsening disease)  **P9 under Follow up of participants and P13 under Research governance, data protection and sponsorship** |
|  | 11c | Strategies to improve adherence to intervention protocols, and any procedures for monitoring adherence (eg, drug tablet return, laboratory tests)  **P7 under Trial intervention 4^th^ paragraph** |
|  | 11d | Relevant concomitant care and interventions that are permitted or prohibited during the trial  **P7 under Trial intervention 5^th^ paragraph** |
| Outcomes | 12 | Primary, secondary, and other outcomes, including the specific measurement variable (eg, systolic blood pressure), analysis metric (eg, change from baseline, final value, time to event), method of aggregation (eg, median, proportion), and time point for each outcome. Explanation of the clinical relevance of chosen efficacy and harm outcomes is strongly recommended  **P7,8 under Outcome Measures** |
| Participant timeline | 13 | Time schedule of enrolment, interventions (including any run-ins and washouts), assessments, and visits for participants. A schematic diagram is highly recommended (see Figure)  **P9 under Outcome Measures, P20 Table 1** |
| Sample size | 14 | Estimated number of participants needed to achieve study objectives and how it was determined, including clinical and statistical assumptions supporting any sample size calculations  **P10 under Sample size considerations** |
| Recruitment | 15 | Strategies for achieving adequate participant enrolment to reach target sample size  **P6 1^st^ paragraph (Ethical considerations section)** |
| **Methods: Assignment of interventions (for controlled trials)** | | |
| Allocation: |  |  |
| Sequence generation | 16a | Method of generating the allocation sequence (eg, computer-generated random numbers), and list of any factors for stratification. To reduce predictability of a random sequence, details of any planned restriction (eg, blocking) should be provided in a separate document that is unavailable to those who enrol participants or assign interventions  **P9 under Randomisation** |
| Allocation concealment mechanism | 16b | Mechanism of implementing the allocation sequence (eg, central telephone; sequentially numbered, opaque, sealed envelopes), describing any steps to conceal the sequence until interventions are assigned  **P9 under Randomisation** |
| Implementation | 16c | Who will generate the allocation sequence, who will enrol participants, and who will assign participants to interventions  **P9 under Randomisation** |
| Blinding (masking) | 17a | Who will be blinded after assignment to interventions (eg, trial participants, care providers, outcome assessors, data analysts), and how  **P9 under Randomisation** |
|  | 17b | If blinded, circumstances under which unblinding is permissible, and procedure for revealing a participant’s allocated intervention during the trial  **(N\A)** |
| **Methods: Data collection, management, and analysis** | | |
| Data collection methods | 18a | Plans for assessment and collection of outcome, baseline, and other trial data, including any related processes to promote data quality (eg, duplicate measurements, training of assessors) and a description of study instruments (eg, questionnaires, laboratory tests) along with their reliability and validity, if known. Reference to where data collection forms can be found, if not in the protocol  **P7,8 under Outcome Measures, P11 and 12 under Economic evaluation and P13 under Data-handling, record keeping and archiving** |
|  | 18b | Plans to promote participant retention and complete follow-up, including list of any outcome data to be collected for participants who discontinue or deviate from intervention protocols  **P6 1^st^ paragraph (Ethical considerations section), P10,11 under Statistical analysis two last paragraphs** |
| Data management | 19 | Plans for data entry, coding, security, and storage, including any related processes to promote data quality (eg, double data entry; range checks for data values). Reference to where details of data management procedures can be found, if not in the protocol  **P13 Data-handling, record keeping and archiving** |
| Statistical methods | 20a | Statistical methods for analysing primary and secondary outcomes. Reference to where other details of the statistical analysis plan can be found, if not in the protocol  **P10,11 under Statistical analysis** |
|  | 20b | Methods for any additional analyses (eg, subgroup and adjusted analyses)  **P10,11 under Statistical analysis** |
|  | 20c | Definition of analysis population relating to protocol non-adherence (eg, as randomised analysis), and any statistical methods to handle missing data (eg, multiple imputation)  **P10,11 under Statistical analysis** |
| **Methods: Monitoring** | | |
| Data monitoring | 21a | Composition of data monitoring committee (DMC); summary of its role and reporting structure; statement of whether it is independent from the sponsor and competing interests; and reference to where further details about its charter can be found, if not in the protocol. Alternatively, an explanation of why a DMC is not needed  **P16 see Data monitoring Committee (DMC) Members** |
|  | 21b | Description of any interim analyses and stopping guidelines, including who will have access to these interim results and make the final decision to terminate the trial  **P11 under Statistical analysis (final paragraph)** |
| Harms | 22 | Plans for collecting, assessing, reporting, and managing solicited and spontaneously reported adverse events and other unintended effects of trial interventions or trial conduct  **P9 under Follow up of participants and Safety Reporting sections** |
| Auditing | 23 | Frequency and procedures for auditing trial conduct, if any, and whether the process will be independent from investigators and the sponsor  **P13 under Research governance, data protection and sponsorship** |
| Ethics and dissemination | | |
| Research ethics approval | 24 | Plans for seeking research ethics committee/institutional review board (REC/IRB) approval  **P5,6 under Ethical considerations, P15 under Declarations Ethics approval and consent to participate** |
| Protocol amendments | 25 | Plans for communicating important protocol modifications (eg, changes to eligibility criteria, outcomes, analyses) to relevant parties (eg, investigators, REC/IRBs, trial participants, trial registries, journals, regulators)  **P15 under Declarations Ethics approval and consent to participate** |
| Consent or assent | 26a | Who will obtain informed consent or assent from potential trial participants or authorised surrogates, and how (see Item 32)  **P6 1^st^ paragraph (under Ethical considerations)** |
|  | 26b | Additional consent provisions for collection and use of participant data and biological specimens in ancillary studies, if applicable **(N\A)** |
| Confidentiality | 27 | How personal information about potential and enrolled participants will be collected, shared, and maintained in order to protect confidentiality before, during, and after the trial  **P13 under Research governance, data protection and sponsorship** |
| Declaration of interests | 28 | Financial and other competing interests for principal investigators for the overall trial and each study site  **P15, 16 under Declarations** |
| Access to data | 29 | Statement of who will have access to the final trial dataset, and disclosure of contractual agreements that limit such access for investigators  **P15 under Declarations** |
| Ancillary and post-trial care | 30 | Provisions, if any, for ancillary and post-trial care, and for compensation to those who suffer harm from trial participation  **P13 under Research governance, data protection and sponsorship final paragraph** |
| Dissemination policy | 31a | Plans for investigators and sponsor to communicate trial results to participants, healthcare professionals, the public, and other relevant groups (eg, via publication, reporting in results databases, or other data sharing arrangements), including any publication restrictions (need to include dissemination section from application  **P14 under Dissemination** |
|  | 31b | Authorship eligibility guidelines and any intended use of professional writers  **N\A** |
|  | 31c | Plans, if any, for granting public access to the full protocol, participant-level dataset, and statistical code  **P15 under Declarations** |
| Appendices |  |  |
| Informed consent materials | 32 | Model consent form and other related documentation given to participants and authorised surrogates  **P28 Appendix 3** |
| Biological specimens | 33 | Plans for collection, laboratory evaluation, and storage of biological specimens for genetic or molecular analysis in the current trial and for future use in ancillary studies, if applicable  **N\A** |

*It is strongly recommended that this checklist be read in conjunction with the SPIRIT 2013 Explanation & Elaboration for important clarification on the items. Amendments to the protocol should be tracked and dated. The SPIRIT checklist is copyrighted by the SPIRIT Group under the Creative Commons “[Attribution-NonCommercial-NoDerivs 3.0 Unported](http://www.creativecommons.org/licenses/by-nc-nd/3.0/)” license.
